# Medication Adherence and Its Association with Health Literacy and Performance in Activities of Daily Livings among Elderly Hypertensive Patients in Islamabad, Pakistan

**DOI:** 10.3390/medicina55050163

**Published:** 2019-05-18

**Authors:** Muhammad Saqlain, Asad Riaz, Muhammad Naeem Malik, Salman Khan, Ali Ahmed, Sohail Kamran, Hussain Ali

**Affiliations:** 1 Department of Pharmacy, Quaid-I-Azam University, Islamabad 45320, Pakistan; msaqlain@bs.qau.edu.pk (M.S.); skhan@qau.edu.pk (S.K.); aliahmed@bs.qau.edu.pk (A.A.); kamran1978pk@yahoo.com (S.K.); 2 Cardiology Department, Pakistan Institute of Medical Sciences (PIMS), Islamabad 44080, Pakistan; dr.asadriaz13@gmail.com (A.R.); drnaeempims@gmail.com (M.N.M.)

**Keywords:** adherence, hypertension, health literacy, elderly

## Abstract

*Background and Objective*: Medication non-adherence is a preventable reason for treatment failure, poor blood pressure control among hypertensive patients and the geriatric population owing to poor physical activity is more vulnerable strata. The objective of this study is to investigate medication adherence and its associated factors among Pakistani geriatric hypertensive patients. *Methods*: A cross-sectional survey-based study was conducted at the out-patient department of the cardiac center from May 2018 to August 2018. A universal sampling technique was used to approach patients and 262 eligible consented patients were interviewed to collect information about socio-demographics, health, and disease-related characteristics using a structured questionnaire. The Morisky Levine Green test was used for the assessment of medication adherence. The Barthel index and single item literacy screener (SILS) was used to measure performance in activities of daily living and health literacy respectively. Chi-square tests and multivariate binary logistic regression analysis were performed to find factors by using SPSS version 20. *Results:* Of the total 262 participants, about 38.9% (*n* = 102) were scored 4 and considered adherent while 61.1% (*n* = 160) were considered as non-adherent. In logistic regression analysis, self-reported moderate (OR = 3.538, *p* = 0.009) and good subjective health (OR = 4.249, *p* = 0.008), adequate health literacy (OR = 3.369, *p* < 0.001) and independence in performing activities of daily living (OR = 2.968, *p* = 0.002) were found to be independent predictors of medication adherence among older hypertensive patients. *Conclusion*: Medication adherence among the older hypertensive population in Pakistan is alarmingly low. This clearly requires patient-centered interventions to overcome barriers and educating them about the importance of adherence.

## 1. Introduction

According to the United Nations (UN) Population Report 2017, the geriatric population is expected to reach 21% by 2050 and out of this 77% will be in developing countries [1]. The rise in the geriatric population in the Asian region is attributed to the current epidemiological transition. According to world factbook, Pakistan has about 9.17 million (4.48%) people above 65 years of age [2] and this figure may escalate to 23.76 million by 2030 and by 2050 the number is expected to reach 43.3 million [3]. As chronic diseases are highly prevalent in the elderly population and there is continuous health deterioration with age, the largest expenditures of medical care are consumed by this population [4].

Hypertension, a silent killer, is a major public health issue across the world with a global prevalence of 31.1% (1.39 billion) [5] and this figure may escalate to 1.56 billion by 2025 [6]. The prevalence of hypertension varies across the globe with the highest rate in Poland (72.5% in females and 68.9% in males) and lowest in rural India (6.8% in female and 3.4% in males) [6]. A recent meta-analysis comprising of 1670 studies revealed that 4–78% of subjects were affected from hypertension [7]. Among all, the highest rates reported in low and middle-income countries rather than high-income countries [7].

The geriatric population is a more vulnerable group as the rate of hypertension increases with age [8]. A large study conducted on 35,125 elderly individuals of age above 50 years from low middle-income countries reported hypertension prevalence ranges from 32.3% in India to 77.9% in South Africa [9]. Hypertension is a major risk factor for a number of common chronic conditions in the older population: ischemic heart disease, stroke, dementia and renal insufficiency [10,11]. Stroke and ischemic heart disease are responsible for greater mortalities in low middle-income countries (LMICs) than high-income countries [12]. The hypertension associated annual number of projected deaths increased from 7.2 million in 1990 to 10.7 million in 2015, a 1.6% increase per year [13].

Pakistan falls in the category of LMICs [14] with heavy population burden of above 200 million (207,774,520) according to national population survey (2017) [15]. The continuous projection of hypertension in a number of individuals shows that it is a challenging health concern in Pakistan [16]. The exact updated data on the nationwide prevalence of hypertension is not available. A recent (2018) meta-analysis of all the published studies regarding the prevalence of hypertension in Pakistan revealed that the pooled prevalence is 23.64% [17]. Prevalence of hypertension in rural central Punjab was highest (60.9%) among individuals of the age group above 60 years [18]. Another recent survey from District Narowal estimated the prevalence of hypertension at 15% among individuals of age group 58–67 years and 6% among individuals aged greater than 68 years [19].

Among various methods of health care, pharmacotherapy is widely practiced and the most common part of the treatment of patients [20]. This can be observed by the fact that nearly 75% of hypertension patients require more than one medicine to control their blood pressure [21]. Adherence to medication is a critical factor in achieving an effective drug therapy and successful management of disease and in chronic diseases like hypertension it becomes more important to adhere to the prescribed regimen [22].

According to the World Health Organization WHO, adherence is “the extent to which a person’s behavior, taking medication, following a diet, and/or executing lifestyle changes, corresponds with agreed recommendations from a health care provider” [23]. It is crucial for treatment success as a study showing antihypertensive medication adherence was significantly associated with better systolic blood pressure control (*p* = 0.001) [24]. Non-adherence, a major health issue, is prevalent in all age groups but its incidence is higher in older individuals, hence they are at higher risk of adverse drug reactions (ADR) and medication mismanagement resulting in poor health outcomes [25]. It has been estimated that among older adults unplanned admissions to hospitals because of adverse drug events is >10% [26].

Researchers are therefore trying to examine the effects of various factors on medication adherence which include age, gender, medication cost, medical status, social support and culture of the society [27]. A number of medications and comorbidities are also considered as important factors that impacted medication adherence [28]. Patient information regarding medication use and intake is highly important especially in older individuals because they face problems in proper understanding of intake time and method [29]. To effectively increase compliance among older adults’ strategies should be formed to properly identify barriers to medication adherence [30]. Various modifiable factors including health literacy, beliefs and patient satisfaction with healthcare services, may be improved to increase medication adherence [31].

Ratzan and Parker (2000) define health literacy as “the degree to which individuals have the capacity to obtain, process, and understand basic health information and services needed to make appropriate health decisions” [32]. These factors significantly affect the proper medication intake ability and disease knowledge in older people [33]. The lower the health literacy, the poorer the ability to understand information regarding health, proper medication intake, understanding prescription, and nutrition tables will be. Health literacy has a strong association with medication adherence and vice versa [34]. Perception about health status is also important as patients with less perceived barriers had better adherence to their medications [35]. In elderly patients, physical activity is also an important aspect in performing routine activities. Patients ability in performing activities of daily living (ADL) is also reported as a factor of medication adherence but the evidence was inconsistent [28,36].

Pakistan has one of the lowest literacy rates among South Asian countries which makes medication adherence a uniquely challenging task. Most patients do not properly understand the prescription hence cannot follow them because it is written in English. Poor regulations in the health industry present another problem as patients take multiple opinions and prescriptions from different health care professionals [37,38]. These factors culminate into poor medication adherence and increased risk of drug-drug interactions and ADRs. From the above discussion, we constructed a hypothesis that there should be an association between medication adherence and patient’s characteristics with health literacy and activities of daily living (ADL) in the geriatric hypertensive Pakistani population.

Thus, the aim of the present study is to investigate the medication adherence rate and to identify its association with socio-demographic, health-related, patient-related and disease-related characteristics in older hypertensive patients.

## 2. Methods

### 2.1. Study Design and Study Setting

This was a cross-sectional, quantitative and descriptive survey-based study. The study was conducted at the out-patient department of cardiac center in a tertiary care hospital located at Islamabad, the capital of the country, namely Pakistan Institute of Medical Science (PIMS).

PIMS is a Government run tertiary care facility and as Pakistan is a developing country where about more than half of population is living at or below the poverty line, the low-cost treatment facilities attract the greater pool of patients. Therefore, it is approached by a population more indicative of the country’s health status.

### 2.2. Sample Size Calculation, Sampling Technique, and Patient Characteristics

The sample size calculated was 260 patients based on 90% power to detect a 20% difference between two groups like male and female at a significance level of 0.05. The sample size was determined by using the PS power and sample size calculator V.3.1.2 [39]. Assuming that by this sample size, it is possible to detect the research hypothesis i.e., difference in medication adherence among different patient categories. Results will be presented as proportion of adherent and non-adherent patients in each category. The study was conducted for a three month period between May 2018 and August 2018. The sampling technique was the universal convenience sampling technique. All the patients who were visiting the out-patient department for their follow up were approached during study duration and eligible patients were invited and requested to participate in the study.

The inclusion criteria to participate in the study were (1) patients aged 65 years or older (2) those who were diagnosed with hypertension and (3) patients who were taking at least one medication for the previous one month. Patients medication record and self-reports were used to ensure diagnosis. Patients with multi-morbidity were also included while patients with cognitive impairment and psychiatric illness, and patients visiting hospital due to exacerbation of acute illness that might lead to hospital admission, were excluded. As per inclusion criteria, all the respondents included in the study were out-patients who were previously on medications, and those patients seeking physician’s checkup as emergency care were excluded.

### 2.3. Data Collection

A structured questionnaire was developed to collect information about (1) socio-demographic characteristics (2) health and medication-related characteristics (3) clinical diagnosis (4) past medication history (5) medication adherence (6) activities of daily living (ADL) and (7) health literacy. Statistical analysis was performed by SPSS Version 20.0 (IBM Corporation, Armonk, NY, USA). The pilot study was also performed to pretest the study instrument in March 2018. This pre-testing showed instrument administration via interview takes 15–25 min and also this is easily understandable for the target population without any cognitive burden. All the interviews of respondents were taken by the principal investigator himself.

#### 2.3.1. Socio-Demographic Characteristics

Socio-demographic characteristics included gender (male, female), age (65–75 76–85, >85 years), Residence (urban, rural), civil status (single, married), education level (illiterate (no schooling), primary (1st–5th class), secondary (6th–13th grade) and tertiary (≥14 grade), employment status (employed, unemployed) and annual income low class (PKR0-299,999), middle class (PKR299,999–999,999), and upper class (≥100,0000). Patients who were divorced or widowed were considered as single. Retired patients who were taking pensions were also considered as employed.

#### 2.3.2. Health and Medication-Related Characteristics

These characteristics involve evaluation of self-reported subjective health (good, medium and poor), smoking status (yes, no), hospital visits in previous 3 months (<2, ≥2), number of times experiencing a fall in the previous one month (none, ≥1), comorbidities (present, absent), number of comorbidities (0–2, >2) and number of medications (1–4, 5–9, ≥10).

#### 2.3.3. Medication Adherence

Medication adherence was assessed by self-reported Morisky Levine Green adherence questionnaire, also referred to as MAQ. It is a four-item questionnaire with high reliability and validity, which has been particularly useful in chronic conditions like hypertension and other cardiovascular diseases. It measures both intentional and unintentional adherence based on forgetfulness, carelessness, stopping the medication when feeling better and stopping the medication when feeling worse [40]. Each item in the questionnaire was responded as 0, Yes and 1, No. The sum of four-items score indicates the level of medication adherence. The score of 4 indicates medication adherence while the score of less than 4 indicates non-adherence. The questionnaire was translated into Urdu according to standard forward-backward translation method and Cronbach alpha calculated was 0.570 which is within the acceptable range (0.45–0.9) [41].

#### 2.3.4. Health Literacy

Single item literacy screener (SILS) developed by Morris et al. was used to determine the health literacy level of study subjects. This is a single item health literacy assessment tool that consists of the question “How often do you need to have someone help you when you read instructions, pamphlets, or other written material from your doctor or pharmacy?”. It is responded as 1—Never, 2—Rarely, 3—Sometimes, 4—Often, and 5—Always. Patients were categorized as having inadequate health literacy if they scored greater than 2 and adequate health literacy if they scored 1 or 2 [42].

#### 2.3.5. Performance in Activities of Daily Living (ADL)

The Barthel index, a 10-item index, was used for assessment of performance in activities of daily living (ADL). The Barthel index is reliable for all users, whether skilled or not. It can assess ADL and mobility in a reliable manner [43]. The score ranges from 0 to 20 and based on score patients were categorized as independent if they scored greater than 16 and dependent if they scored less than 16 [44].

### 2.4. Ethical Approval

The study was performed in accordance with the declaration of Helsinki. The study was approved by an Ethical review board of PIMS hospital (1-1/2015/ERB/SZABMU/229, dated: 25/4/18). Patients were invited to participate in the study and those who agreed were told about the nature and purpose of the study, and patients officially consented both orally and in writing.

### 2.5. Statistical Analysis

Statistical analysis was performed by SPSS version 20 (IBM, Armonk, NY, USA). Descriptive statistics were calculated as frequencies and percentages were calculated for categorical variables. Chi-square tests were used to determine the difference in adherence by sample characteristics. Multivariate binary logistic regression analysis was performed to determine factors of medication adherence by using variables of *p* < 0.1 in univariate analysis. Results are expressed as ORs accompanied by 95% CIs, and *p* < 0.05 was used for statistical significance.

## 3. Results

### 3.1. Sociodemographic, Health- and Medication-Related Characteristics of Participants

To ensure the desired sample size, a total of 290 patients were approached out of which 262 consented patients (response rate = 90.34) were included in the study according to inclusion criteria.

About 64.5% (*n* = 169) of participants were females, 84.7% (*n* = 222) were aged 65–75 years and more than half (55.7%, *n* = 146) were illiterate. About half (53.8%, *n* = 141) of the individual was taking 5–9 different medications and about 75.6% were reported to have less than two co-morbid conditions. A total of 37.4% (*n* = 98) had adequate health literacy and the majority (71.0%, *n* = 186) of patients were independent in performing activities of daily living (Table 1). The common comorbid conditions were coronary artery disease (53.43%), diabetes (28.6%), valvular heart disease (13.7%) and heart failure (12.2%) among elderly hypertensive patients (Table 2).

### 3.2. Difference in Medication Adherence by Patient Characteristics

Of the total 262 participants, about 38.9% (*n* = 102) were scored 4 and had high adherence while 61.1% (*n* = 150) were (scored <4) considered to be non-adherent (Table 1). Chi-square tests were employed to determine the difference in medication adherence and patient characteristics. The statistically significant difference was observed between adherence and self-reported subjective health, comorbidity status, number of comorbidities, health literacy and activities of daily living. Results revealed that adherence significantly differs from self-reported health (χ^2^ = 7.697, *p* = 0.021). We also found that adherence significantly differs with the presence of comorbidity (χ^2^ = 4.022, *p* = 0.045) and highly associated with a number of comorbidities (χ^2^ = 4.093, *p* = 0.043). High adherence was also highly associated with independence in daily living activity than dependence in daily living activity (χ^2^ = 16.590, *p* < 0.001) and individuals with adequate health literacy were more adherent to their medications than individuals with inadequate health literacy (χ^2^ = 24.356, *p* < 0.001). While, the results showed that no statistically significant difference was found between adherence and gender, age, marital status, education level, annual income, employment status, residence, smoking status, hospital visits, number of falls experienced and number of medications as *p* > 0.05 (Table 3).

### 3.3. Factors Affecting Medication Adherence

Multivariate binary logistic regression analysis was performed to find determinants of medication adherence among older adults with hypertension. Independent variables with *p* < 0.1 in the primary analysis was employed in the regression model. Results revealed that patients who thought their health was moderate had 3.538 times more adherence (OR = 3.538, *p* = 0.009) while in individuals with the perception of good subjective health the odds of adherence increased by 4.249 times (OR = 4.249, *p* = 0.008) compared to individuals who reported poor subjective health. Additionally, the study revealed that health literacy and activities of daily living were significant predictors of medication adherence. Older adults with adequate health literacy were 3.369 times more adherent (OR = 3.369, *p* < 0.001) compared to elderly patients with inadequate health literacy. Geriatric hypertensive patients who were independent in performing daily living activities had 2.968 times more adherence (OR = 2.968, *p* = 0.002) compared to those who were dependent on performing daily living activities (Table 4).

## 4. Discussion

The current study was conducted to investigate medication adherence and associated factors among older hypertensive patients. Results revealed that 38.9% of participants were adherent while the majority of participants (61.1%) were non-adherent to their medications. Adequate health literacy and independence in the performance of daily living activities were found as statistically significant factors that affect medication adherence.

The rate of medication adherence found in the present study was consistent with the finding of different studies, as literature reported the prevalence of adherence in older adults ranged from 26% to 59% [45]. A recent (2017) meta-analysis reported a global prevalence of non-adherence was 41% [46]. The results of this study are consistent with a recent study conducted on 183 older hypertensive patients (33.3%) in Cameron [47]. Another study had reported a similar adherence rate (34.2%) among 585 older Chinese hypertensive patients [48]. Adherence was measured by Morisky medication adherence scale (MMAS-8 item) in both of these studies. The result of this study is compared to Cameroon and Chinese studies owing to the fact that Cameroon is also a low-middle income nation while China is a developing middle-income nation as well as neighboring country [49].

In contrary to the findings of this study, higher medication adherence was reported among older hypertensive patients in Korea (41%) [50], Hong Kong (44.1%) [51], Boston (52%) [36] and Spain (65.7%) [28]. The Morisky Green Levine scale was used for assessment of adherence rate in these studies. The higher adherence rate might be due to higher literacy, well-managed health care facilities and high availability of medicines in developed countries compared to developing countries [52]. A significantly higher adherence rate in Spain might be because of the fact that study participants were homebound elderly hypertensive individuals [28].

There is a wide variation in adherence rate depending upon the population studied, method of measurement and definition of adherence [53]. The low medication adherence rate reported in the present study is a current snapshot of this important issue in the Pakistani population. This low adherence rate might be due to the fact that the majority of study participants were illiterate, had a low annual income, were unemployed and taking multiple medications. From the perspective of the country’s health system, the following reasons might be attributed to the low medication adherence rate. Higher illiteracy, a poorly regulated health care system and varying opinions of health care physicians may contribute to medication non-adherence [54]. Over-burdened health care setup might also lead towards non-adherence due to unavailability of proper counseling time for patients while no special health packages were available for elderly citizens [55]. Other reasons might include high price, the poor availability of essential medicines and no insurance policy that might be more helpful for older individuals [56].

The study findings showing that adherence was different among patients who had the comorbid condition and also changed by a number of comorbidities (*p* < 0.05). It is postulated that the presence of concomitant diseases influences the attitude and perception of being helpless and sick among older adults that leads to strict medication-taking behavior [57]. The results of this study were consistent with the findings of Berry et al. (*p* = 0.02) [36] and Wang et al. (*p* < 0.001) [58]. On contrary to these findings, no such association was found in a Korean study (*p* = 0.918) [50].

After logistic regression analysis, self-reported health found as a significant factor of medication adherence. Patients who thought that their health was moderate and good had a higher adherence rate than those who reported poor subjective health. A strong association of self-perceived health status with medication adherence was also observed in chi-square analysis. This result is in line with a previous study which reported a strong association (*p* = 0.012) between medication adherence and self-reported health [59].

The ability to perform activities of daily living (ADL) found as another important factor that was significantly associated with medication adherence in univariate chi-square analysis. Independence in doing activities of daily living is also found as an important predictor of medication adherence in multivariate regression analysis. The results are consistent with findings of a Spanish study where dependence on doing daily living activities was a significant predictor of non-adherence [28]. On contrary to findings of this study, Berry reported no association between the capacity of doing ADL and medication adherence (*p* = 0.09) [36]. A possible explanation is that patient who is able to perform their activities independently might be more active in taking care of themselves, a better understanding of their medications as better functional status is also an indication of good physical and mental health. Self-care is important for the optimal health of elderly individuals [60].

The present study also provides insight into the relationship between health literacy and medication adherence among the geriatric hypertensive population. Health literacy was found as the strongest predictor of medication adherence in both chi-square analysis and multivariate logistic regression analysis. Among patient-related characteristics that affect medication adherence, health literacy is considered as the most important modifiable factor [31]. Different researchers assessed the relationship between medication adherence and health literacy under the assumption that health literacy is an important factor in medication adherence. In line with previous studies [59,61], our study also showed that patients with adequate health literacy are more adhered to their medications compared to patients with inadequate health literacy. By contrast, Kripalani found unexpectedly low odds of non-adherence among patients with inadequate health literacy [62].

Health literacy is an important issue of health care today. In older adults, poor health literacy regarding the use of medication may be a significant factor of premature mortalities independent of socioeconomic status, comorbidities, cognitive ability and education [63]. In an English study, mortality risk increased by 26% in patients with low health literacy compared to patients who could understand instructions regarding medication utilization efficiently [63]. Lower health literacy is a common issue among geriatric patients and associated with poor adherence and poor health outcomes. From the patient viewpoint, health literacy is directly related to the effective understanding of health issues which leads to the accurate identification of health problems and subsequent management of the issue in a better way [64].

### 4.1. Strengths and Limitations

To best of our knowledge, this is the first study that investigated medication adherence among geriatric hypertensive patients in Pakistan. Additionally, this study provides insight into health literacy status and its association with medication adherence along with other factors. While previous studies had a broader spectrum with no special focus on older patients. This study provides an opportunity to address the underlying barriers of medication adherence and highlights the prime need for the development of interventions and policies that improve health literacy and patients’ living status.

The study also has some limitations. First, as this is a single center, the cross-sectional study comprised of a small number of patients. Second, medication adherence is measured by a self-reported measure though it is a widely used validated tool; though other methods like pill count are much more accurate in measure. Moreover, lower reliability coefficients (0.57) in the present study also limit its validation and reliability. Third, health literacy was measured by a single question. Although, this is a validated tool and was used with the intention to minimize the cognitive burden on patients due to a lengthy but less accurate questionnaire than other standard tools like Test of Functional Health Literacy.

### 4.2. Practice Implications

This study is showing poor medication adherence among older hypertensive patients that provides an opportunity to address barriers to medication adherence among the vulnerable patient population. The results of the present study have a number of implications. Elderly patients with good subjective health, are independent in performing activities of daily living and adequate health literacy are more adherent to their medications. Thus, lower medication adherence rate can be addressed by implementing effective patient-centered intervention with a prime focus on modifiable factors that affect medication adherence like health literacy, patient beliefs, and patient-physician communication [4]. The World Health Organization (WHO) made it mandatory for the policymakers to improve health literacy at the population level, health care professional must be vigilant to identify patient health literacy and measures should be taken to overcome the barriers for improvement in health literacy and thus medication adherence [64]. A collective approach by doctors, pharmacists and nurses is the need of the hour to enhance health literacy of the population and adherence to medications [65].

## 5. Conclusions

This study demonstrated that medication adherence among the older hypertensive population in Pakistan is alarmingly low and is similar to global findings. The finding also revealed that health literacy and activities of daily living are significant factors in medication adherence. This may be attributed to low literacy rate, lack of patient-centered education and counseling by health care providers. This clearly requires patient-centered interventions to overcome barriers and educating them about the importance of adherence. It is also recommended that future studies with more focus on modifiable risk factors of medication adherence and its impact on blood pressure control should be performed.

## Figures and Tables

**Table 1:** Sociodemographic, health- and medication-related characteristics of participants.

Variables	Female	Male	Total
	*n* = 169	*n* = 93	*n* = 262
	*n* (%)	*n* (%)	*n* (%)
**Age**			
65–75	149 (56.9)	73 (27.9)	222 (84.7)
76–85	13 (5.0)	16 (6.1)	29 (11.1)
>85	7 (2.7)	4 (1.5)	11 (4.2)
**Marital status**			
Single/widowed/divorced	52 (19.8)	2 (3.7)	54 (20.6)
Married	117 (44.7)	91 (34.7)	208 (79.4)
**Education level**			
None	119 (40.5)	27 (10.3)	146 (55.7)
Primary	38 (14.5)	30 (11.5)	68 (26.0)
Secondary	5 (1.9)	29 (11.1)	34 (13.0)
Tertiary	7 (2.7)	7 (2.7)	14 (5.3)
**Annual income**			
Low class	93 (35.5)	47 (33.6)	140 (53.4)
Middle class	53 (20.2)	36 (13.7)	89 (34.0)
Upper class	23 (8.8)	10 (3.8)	33 (12.6)
**Employment status**			
Employed	48 (18.3)	64 (24.4)	112 (42.7)
Unemployed	121 (46.2)	29 (11.1)	150 (57.3)
**Residence**			
Urban	92 (35.1)	54 (20.6)	146 (55.7)
Rural	77 (29.4)	39 (14.9)	116 (44.3)
**Self-reported health**			
Poor	25 (9.5)	12 (4.6)	37 (14.1)
Moderate	105 (40.1)	68 (26.0)	173 (66.0)
Good	39 (14.9)	13 (5.0)	52 (19.8)
**Number of hospital visits in previous 3 months**			
Less than 2	104 (39.7)	42 (16.0)	146 (55.7)
2 or More than 2	65 (24.8)	51 (19.5)	116 (44.3)
**Number of hospital admission in 6 months**			
None	152 (58.0)	66 (25.2)	218 (83.2)
1 or More than 1	17 (6.5)	27 (10.3)	44 (16.8)
**Number of times fall experienced in previous 1 month**			
None	163 (62.2)	74 (28.2)	237 (90.5)
One or more times	6 (2.3)	19 (7.3)	25 (9.5)
**Number of medications**			
1–4	62 (23.7)	33 (12.6)	95 (36.3)
5–9	88 (33.6)	53 (20.2)	141 (53.8)
10 or more	19 (7.3)	7 (2.7)	26 (9.9)
**Comorbidity?**			
Present	115 (43.9)	51 (19.5)	166 (63.4)
Absent	54 (20.6)	42 (16.0)	96 (36.6)
**Number of comorbidities**			
0–2	129 (49.2)	72 (27.5)	201 (76.7)
>2	40 (15.3)	21 (8.0)	61 (23.3)
**Performance of ADL**			
Dependent	38 (14.5)	38 (14.5)	76 (29.0)
Independent	131 (50.0)	55 (21.0)	186 (71.0)
**Health Literacy**			
Inadequate	121 (46.2)	43 (26.2)	164 (62.6)
Adequate	48 (18.3)	50 (19.1)	98 (37.4)
**Medication Adherence**			
Adherent	70 (26.7)	32 (12.2)	102 (38.9)
Non-adherent	99 (37.8)	61 (23.3)	160 (61.1)

Abbreviation: ADL, Activities of daily living.

**Table 2:** Comorbid conditions among elderly patients.

Indications	Female (*n* = 169) *n* (%)	Male (*n* = 93) *n* (%)	Total (*n* = 262) *n* (%)
**Hypertension**	**169 (64.5)**	**93 (33.5)**	**262 (100)**
**Comorbid conditions**			
Coronary artery disease	128 (75.7)	12 (12.9)	140 (53.43)
Diabetes	50 (29.5)	25 (26.8)	75 (28.6)
Valvular heart disease	21 (12.4)	15 (16.1)	36 (13.7)
Heart failure	15 (8.8)	17 (18.2)	32 (12.2)
Osteoarthritis	3 (1.7)	2 (2.2)	5 (1.9)
Asthma/COPD	6 (3.5)	5 (5.3)	11 (4.1)
Dyslipidemia	3 (1.7)	11 (11.8)	14 (5.3)
HCV	3 (1.7)	2 (2.2)	5 (1.9)
CKD	1 (0.6)	5 (5.3)	6 (2.3)
Others (GIT, CNS etc.)	13 (7.7)	8 (8.6)	21 (8.1)

Abbreviations: CKD: Chronic kidney disease, COPD: Chronic obstructive pulmonary disease, HCV: Hepatitis C virus, GIT: Gastrointestinal tract, CNS: Central nervous system.

**Table 3:** Difference in medication adherence by sample characteristics.

	Non-Adherent	Adherent	χ^2^	*p*-Value
	*n* (%)	*n* (%)		
	160 (61.1)	102 (38.9)		
**Gender**			1.240	0.265
Female	99 (37.8)	70 (26.7)		
Male	61 (23.3)	32 (12.2)		
**Age**			1.631	0.442
65–75	132 (50.4)	90 (34.4)		
76–85	20 (7.6)	9 (3.4)		
>85	8 (3.1)	3 (1.1)		
**Marital status**			0.384	0.536
Single/widowed/divorced	31 (11.8)	23 (8.8)		
Married	129 (49.2)	79 (30.2)		
**Education level**			3.897	0.273
None	95 (36.3)	51 (19.5)		
Primary	41 (15.6)	27 (10.3)		
Secondary	16 (6.1)	18 (6.9)		
Tertiary	8 (3.1)	6 (2.3)		
**Annual income**			3.730	0.155
Low class	92 (35.1)	48 (18.3)		
Middle class	52 (19.8)	37 (14.1)		
Upper class	16 (6.1)	17 (6.5)		
**Employment status**			0.024	0.877
Unemployed	91 (34.7)	59 (22.5)		
Employed	69 (26.3)	43 (16.4)		
**Residence**			0.046	0.830
Urban	90 34.4)	56 (21.4)		
Rural	70 (26.7)	46 (17.6)		
**Self-reported subjective health**			7.697	**0.021**
Poor	30 (11.5)	7 (2.7)		
Moderate	102 (38.9)	71 (27.1)		
Good	28 (10.7)	24 (9.2)		
**Smoking status**			0.879	0.349
Non-smokers	131 (50.0)	88 (33.6)		
Smokers	29 (11.1)	14 (5.3)		
**Number of hospital visits in previous 3 months**			1.126	0.289
Less than 2	85 (32.4)	61 (23.3)		
2 or More	75 (28.6)	41 (15.6)		
**Number of times fall experienced in previous 1 month**			0.299	0.585
None	146 (55.7)	91 (34.7)		
1 or more times	14 (5.3)	11 (4.2)		
**Number of medications**			2.783	0.249
1–4	52 (19.8)	43 (16.4)		
5–9	90 (34.4)	51 (19.5)		
10 or more	18 (6.9)	8 (3.1)		
**Comorbidity?**			4.022	**0.045**
Present	109 (41.6)	57 (21.8)		
Absent	51 (19.5)	45 (17.2)		
**Number of comorbidities**			4.093	**0.043**
<2	116 (44.3)	85 (32.4)		
>2	44 (16.8)	17 (6.5)		
**ADL (Barthel index score)**			16.590	**<0.001**
Dependent	61 (23.3)	15 (5.7)		
Independent	99 (37.8)	87 (33.2)		
**Health Literacy**			24.356	**<0.001**
Inadequate	119 (45.4)	45 (17.2)		
Adequate	41 (15.6)	57 (21.8)		

Abbreviation: ADL, Activities of daily living; χ^2^, Chi square; Note: Bold values show statistically significant difference.

**Table 4:** Multivariate logistic regression analysis to find factors of medication adherence.

Characteristics	OR	95% CI	*p*-Value
**Self-reported subjective health**			
Poor	1.0	-	-
Moderate	3.538	1.366–9.163	**0.009**
Good	4.249	1.452–12.435	**0.008**
**Comorbidity?**			
Absent	1.0	-	-
Present	1.522	0.822–2.820	0.181
**Number of comorbidities**			
<2	1.0	-	-
>2	1.183	0.552–2.531	0.666
**Performance of ADL**			
Dependent	1.0	-	-
Independent	2.968	1.1506–5.851	**0.002**
**Health Literacy**			
Inadequate	1.0	-	-
Adequate	3.369	1.905–5.959	**<0.001**

Abbreviation: ADL = Activities of daily living, OR: Odds ratio, CI: Confidence interval; Note: Bold values show statistically significant factors.

## Abbreviations

ADR	Adverse drug reaction
ADL	Activities of daily living
LMICs	Low-middle income countries
MAQ	Medication adherence questionnaire
PIMS	Pakistan Institute of Medical Sciences
SILS	Single item literacy screener
US	United States
WHO	World Health Organization
